# Phacotrabeculectomy versus Phaco with Implantation of the Ex-PRESS Device: Surgical and Refractive Outcomes—A Randomized Controlled Trial

**DOI:** 10.3390/jcm10030424

**Published:** 2021-01-22

**Authors:** Joanna Konopińska, Anna Byszewska, Emil Saeed, Zofia Mariak, Marek Rękas

**Affiliations:** 1 Department of Ophthalmology, Medical University of Białystok, M. Sklodowska-Curie 24A STR, 15-276 Białystok, Poland; emilsaeed1986@gmail.com (E.S.); mariakzo@umb.edu.pl (Z.M.); 2 Department of Ophthalmology, Military Institute of Medicine, Szaserów 128 STR, 04-141 Warszawa, Poland; ania.byszewska@gmail.com (A.B.); rekaspl@gmail.com (M.R.)

**Keywords:** astigmatism, glaucoma, intraocular pressure, phacotrabeculectomy, Ex-PRESS device

## Abstract

The aim of this study was to compare surgical and refractive outcomes between phacotrabeculectomy (P-Trab) and phaco with Ex-PRESS (P-Ex-PRESS) for glaucoma at a 6-month follow-up. This prospective randomized controlled trial included 81 eyes; 43 eyes (53%) and 38 eyes (47%) were assigned to the P-Ex-PRESS and P-Trab groups, respectively. Refraction, intraocular pressure (IOP), and best-corrected visual acuity were measured. Refractive change was analyzed using the cylinder’s magnitude, and polar analysis assessed the change in the trend of astigmatism [with-the-rule, against-the-rule (ATR), oblique (OBL)], evaluating mean astigmatism in centroid form. All patients showed a statistically significant postoperative decrease in IOP (*P* < 0.05). There were no differences between the groups in terms of postoperative IOP and visual outcomes or in astigmatism preoperatively or postoperatively (*P* = 0.61, *P* = 0.74). In both groups, the mean preoperative and postoperative astigmatism were ATR and OBL, respectively. Preoperative and postoperative centroids in the P-Ex-PRESS group were 0.44 ± 1.32 D at 177° and 0.35 ± 1 D at 8°, respectively, (*P* = 0.5) and in the P-Trab group were 0.16 ± 1.5 D at 141° and 0.39 ± 1.38 D at 29°, respectively (*P* = 0.38). Both P-Ex-PRESS and P-Trab showed comparable antihypertensive efficacy in treating open-angle glaucoma over 6 months. Preoperative and postoperative astigmatism did not differ between groups. The groups showed comparable results for final visual acuity.

## 1. Introduction

Trabeculectomy and implantation of the Ex-PRESS mini device (Alcon Laboratories, Fort Worth, TX, USA) are anti-glaucoma procedures that improve subconjunctival outflow [1]. Although trabeculectomy remains the “gold standard” for anti-glaucoma surgery, implantation of the less-invasive Ex-PRESS mini seton is also effective [2]. Both procedures establish a new, alternative outflow route within the trabecular meshwork and evacuation of the aqueous humor via artificial filtering fistulae. They can be used as a standalone procedure or combined with phacoemulsification when patients have both glaucoma and cataract. A simultaneous approach can reduce the anesthesia and surgery time, with less recovery time and reduced overall cost of care to the patient and the health system [3]. Penetrating glaucoma procedures require extended ocular tissue interference, such as conjunctival preparation, scleral cutting, and suture application. However, they provide high hypotensive efficacy, and are reasonable treatments for moderate to advanced glaucoma, eliminating the chronic need for intraocular pressure (IOP)-lowering eye drops. Patients undergoing glaucoma surgery often have a brief period of reduced visual acuity in the early postoperative period, which can persist on a long term basis in some cases [4]. This could be due to surgically induced astigmatism (SIA) after penetrating glaucoma procedures such as trabeculectomy [5,6,7]. The degree of astigmatism may be influenced by the surgical technique (i.e., scleral flap size), the sutures used for conjunctival closure (mattress vs. knotted), suture tightness, use of cauterization, the cauterization technique (dry vs. wet), the duration of surgery, the trabeculectomy tool used (punch vs. knife), and use of anti-metabolites (dose and time) [7]. Moreover, IOP fluctuations in the postoperative period, caused by the pressure of the upper eyelid on the filtering bleb, may also induce astigmatism [7,8]. With-the-rule (WTR) astigmatism after phacotrabeculectomy (P-Trab) can worsen postoperative visual acuity [9,10]. Implantation of Ex-PRESS device combined with phaco (P-Ex-PRESS) differs in that it does not require cutting a fragment of the sclera, limbus, or iris or the use of a punch. Some authors report that this may reduce wound gape and “sinking” due to tissue removal [3]. Therefore, it remains unclear whether P-Ex-PRESS surgery generates less astigmatism than P-Trab surgery. Several studies have assessed SIA after trabeculectomy, non-penetrating surgery, or minimally invasive glaucoma surgery, with most studies obtaining data through a retrospective chart review without a control group. There is no clear data on refractive outcomes of Ex-PRESS device surgery, and no study has evaluated the refractive outcomes of combined Ex-PRESS implants and cataract surgery versus P-Trab in a prospective design Our study aimed to compare the amount of SIA and refractive change in P-Trab versus P-Ex-PRESS surgery to evaluate the relative surgical success of these procedures in a randomized, controlled trial with a 6-month follow-up. This research contributes much-needed data on the efficacy and safety profile of the Ex-PRESS device in comparison with trabeculectomy.

## 2. Materials and Methods

### 2.1. Patients

This study adheres to the tenets of the Declaration of Helsinki and the Principles of Good Clinical Practice developed by the European Union. The study protocol was approved by the Bioethics Committee at the Medical University in Białystok under the number R-I-002/443/2014 and registered on clinicaltrials.gov (registration number: NCT04335825). Written informed consent to participate for at least 6 months was obtained from all patients after an explanation of the nature of the procedure and surgical alternatives. The study protocol was similar to that documented in our previous work [11].

We recruited consecutive patients who were referred to the ophthalmology clinic of our hospital and qualified for combined surgery. Glaucoma with coexisting cataract graded NC1 or NC2 by the Lens Opacities Classification System III scale was the surgical indication. Patients with primary open-angle glaucoma, pseudoexfoliation glaucoma, and pigmentary glaucoma, where target IOP was not achieved, despite maximally-tolerated topical and systemic medication and well-documented visual field defect progression, were eligible for treatment. A patient qualified for surgery if any of the following additional inclusion criteria was present: significant diurnal variations in IOP, poor patient compliance, or allergy to topical anti-glaucoma drugs with progressive visual field loss. The exclusion criteria were as follows: lack of consent for study participation, history of eye surgery or laser procedures within the eye, closed or narrow-angle glaucoma, diabetes, advanced macular degeneration, and active inflammatory disease.

The randomized prospective study included 81 eyes of 81 patients. A computer-generated randomization list was created, with allocation concealment. Randomization was performed using sealed envelopes, opened on the day of surgery, to determine the randomization group. The eyes were individually randomized in a 1:1 ratio to either phacoemulsification with simultaneous Ex-PRESS mini glaucoma shunt implantation (43 eyes (53%)) or P-Trab (38 eyes (47%)).

### 2.2. Preoperative Examination

Detailed data on patient demographics (age, sex), previous treatments, and surgical procedures were collected at the time of qualification. Before surgical treatment, all patients underwent a basic examination, which included determination of IOP, uncorrected distance visual acuity, best-corrected visual acuity (BCVA), and refractive findings (refractive error: sphere and cylinder with axis), axial length, and biomicroscopic examination of the anterior and posterior eye segments, with a detailed assessment of the retina and optic nerve disc. The BCVA was examined with the Snellen notification and expressed as logMAR units. A Snellen BCVA of 1.0 (100% or 20/20) equals a logMAR of 0.

The intraocular lens (IOL) power was calculated with the IOL Master 700 (Carl Zeiss Meditec, AG, Jena, Germany). All patients were implanted with the same type of IOL. Gonioscopy was performed along with the field of vision test( Humphrey Field Analyzer, program SITA standard 24-2, Carl Zeiss-Humphrey Systems, Dublin, CA, USA). The IOP was measured during preoperative examination using a slit-lamp–mounted Goldmann applanation tonometer in accordance with the Advanced Glaucoma Intervention Study. The reading in mm Hg was rounded to the closest integer. Each measurement was repeated twice, and if the difference between the two readings was ≥3 mm Hg, a third measurement was taken. The mean of two or three measurements was used to determine the IOP.

### 2.3. Surgical Technique

All surgical procedures were performed under retrobulbar anesthesia (2% xylocaine and 0.5% bupivacaine) by the same experienced surgeon (JK) for the duration of the study. In both procedures, the fornix-base conjunctiva was dissected, and the sclera was exposed. A limbus-based, square-shaped (4 mm × 4 mm) scleral flap was dissected using the technique described by Traverso et al. [1]. A clear corneal incision of 2.25 mm was made temporally with the phaco-chop technique for phacoemulsification using the Megatron S4 HPS (Geuder, Heidelberg, Germany), and an IOL was implanted into the capsular bag. The same type of IOLs (Akreos Adapt, Bausch & Lomb, Rochester, NY, USA) were implanted for all surgeries. In the P-Ex-PRESS group, a mini glaucoma shunt was implanted at the one o’clock position, using a technique described previously [12]. In the P-Trab group, iridectomy was made at the same position. The scleral flap was closed with 10/0 nylon sutures (four knotted sutures) and the conjunctival closure was achieved with absorbable sutures. During trabeculectomy and Ex-PRESS mini glaucoma shunt implantation, 5-fluorouracil (5-FU; 50 mg/mL) was used on a standard basis and applied to the scleral wound bed for 3.5 min to avoid contact with the conjunctival incision area [9].

### 2.4. Postoperative Protocol

During the follow-up visits, the IOP and BCVA were measured, as mentioned above, by the same unmasked resident doctor. Detailed biomicroscopic evaluation of the anterior chamber and fundus of the eye was performed. During the postoperative assessment, both complications and the number of IOP-lowering medications administered were documented. Additional procedures were performed when insufficient filtration was noted as an elevated IOP (≥16 mm Hg) or an underdeveloped or completely flat filtering bleb [9]. Inadequate filtration was diagnosed during the first 2 weeks after surgery when healing did not yet restrict subconjunctival outflow and a progressive increase in the IOP >16 mm Hg was observed [9]. Patients were examined for the presence and proper functioning of the filtering bleb and the development of subconjunctival fibrosis (observed as engorged and tortuous blood vessels above the scleral flap). Needling was performed based on a diagnosis of fibrosis (based on the above clinical signs), insufficient subconjunctival outflow, increase in IOP, or flattening of the bleb, by the same examiner throughout the whole follow-up. If fibrosis occurred, 5-FU subconjunctival injections were administered (5 mg in 0.2 mL), combined with needling when appropriate, for 5 consecutive days or until fibrosis disappeared and the IOP stabilized, provided that no anti-metabolite-related adverse effects were encountered [10]. Needling was performed when a flat, dysfunctional filtering bleb was observed. Suture lysis was performed within the first 2 weeks after surgery when poor filtration through the bleb was observed (due to overly tight suturing of the scleral flap). Needling and suture lysis were not considered as failures. An IOP ≤6 mm Hg was defined as ocular hypotony.

The success rate was defined as either complete or qualified. Complete surgical success was defined as IOP ≤18 mm Hg without anti-glaucoma medications, whereas qualified success was defined as IOP ≤18 mm Hg with a maximum of two anti-glaucoma medications (determined by the number of active ingredients). In the case of IOP >18 mm Hg with or without anti-glaucoma medications or when the eye required further surgical intervention, surgery was considered a failure. No anti-glaucoma medications were allowed on the day of the operation. When surgery did not achieve the expected results, medications were re-administered as recommended by the European Glaucoma Society rules. 

Control examinations were performed both before treatment and on the first and seventh postoperative days, as well as at postoperative days 30, 90, and 180. Postoperatively, all eyes were treated with topical postoperative steroids (Loteprednol, one drop three times daily for 4 weeks), and tapered to twice daily after 1 week. Antibiotic (Moxifloksacin) was administered as a single drop three times daily for 2 weeks and nonsteroidal anti-inflammatory drugs as one drop three times daily for 4 weeks.

### 2.5. Refractive Data Evaluation

Autorefractometry data from before and 180 days after surgery were included in the analysis. Refractive and vector analyses were performed. Refractive analysis, a simple arithmetic calculation of the mean of the cylinder without considering its axis, was performed to compare with the numerical results available in the literature. This difference in the mean value of the cylinder allows for reporting the mean change in the magnitude of astigmatism. All calculations were performed using the plus form of the cylinder.

The vector analysis is a calculation of the cylinder change considering its axis. The preoperative and postoperative refractive measurements (cylinder with its axis) were evaluated by vector analysis, according to the method proposed by Holladay et al. [13]. Data were converted from standard polar values (cylinder and axis) to Cartesian values (points with x, y coordinates) to evaluate trends in astigmatism (against-the-rule [ATR], WTR, or oblique) and to define mean astigmatism in the centroid form.

For conversion from polar to Cartesian values, the following mathematical formulae were applied:

x = cyl × cos (2 × axis)

y = cyl × sin (2 × axis).

Cartesian coordinates were converted to standard polar values using the below formulae:cyl = √(x^2^ + y^2^)angle = ½ × [tan^−1^ (y/x)]if x > 0 and y > 0→axis = angleif x < 0→axis = angle + 90°if x > 0 and y < 0→axis = angle + 180°if x = 0 and y < 0→axis = 135°if x = 0 and y > 0→axis = 45°if x = 0 and y = 0→axis = 0°if y = 0 and x < 0→axis = 90°if y = 0 and x > 0→axis = 0°.

This calculation was performed for each individual to obtain the surgically induced refractive change. The mean of all x and y values were calculated to allow calculation of the aggregate refractive change of the analyzed groups. Data were displayed as double-angle plots because the angles were doubled owing to the return of the astigmatism vector to the same value when traversing 180°.

The major and minor axes of the centroid of the ellipse were determined by calculating the standard deviations of the x and y coordinates. The astigmatism trend was evaluated based on the shape factor and the centroid axis.

The aggregate data considered the mean value of astigmatism (mean magnitude of the cylinder), which does not include the axis analysis or centroid calculation.

The centroid was used to define the direction of astigmatism (WTR, against-the-rule, or oblique).

The double-angle plots were prepared using the double-angle-plot-tool for astigmatism available on the American Society of Cataract and Refractive Surgery website. Data on astigmatism values were depicted in cumulative data plots at 0.5-D intervals.

### 2.6. Statistical Evaluation

In both groups, quantitative data are expressed as arithmetic means, standard deviations, and medians. Qualitative characteristics are expressed as numbers and percentages. Data were tested for normality using the Shapiro–Wilk test. Between-group comparisons were made using Student’s *t*-test or Mann-Whitney U test. The x^2^ test of independence for two variables was used to compare quantitative characteristics. Values of *P* < 0.05 were considered to indicate statistical significance. Analyses were conducted using SPSS version 24.0 (IBM, Armonk, NY, USA).

## 3. Results

A total of 43 and 38 patients underwent P-Ex-PRESS and P-Trab procedures, respectively. The demographic data are summarized in Table 1.

### 3.1. Intraocular Pressure

The mean IOP levels before and after surgery are summarized in Table 2.

### 3.2. IOP-Lowering Drugs

The average numbers of IOP-lowering medications before and after surgery are summarized in Table 3. In the P-Ex-PRESS and P-Trab groups, two drugs were used before surgery in 20.6% (*n* = 8) and 22.6% (*n* = 8) of patients, respectively. However, during the follow-up, 50% (*n* = 21) and 75% (*n* = 28) of patients were drug-free in the P-Ex-PRESS and P-Trab groups, respectively.

### 3.3. Surgical Success

By the criterion of pressure ≤18 mm Hg, 44% (*n* = 19) and 49% *(n* = 18) of patients achieved complete success in the P-Ex-PRESS and P-Trab groups, respectively (*P* = 0.681). Qualified success was achieved in 63% (*n* = 27) of patients in the P-Ex-PRESS group and 71% (*n* = 27) of patients in the P-Trab group (*P* = 0.561).

### 3.4. Best-Corrected Visual Acuity

The preoperative and postoperative BCVAs are shown in Table 4.

In the P-Ex-PRESS group, BCVA was one line worse in four patients (8.7%). In 12 patients (15.2%), BCVA remained at baseline after surgery and improved from one to nine lines in 61 (76.1%) patients. Vision deterioration due to posterior capsular-bag opacification was noted in one patient. Macular edema caused by chronic hypotension was observed in a single case during the follow-up.

In the P-Trab group, BCVA decreased by one Snellen line in seven patients (8.8%), remained unchanged in six (7.7%), and increased by one to nine lines in 68 (83.5%) patients. The reasons for vision deterioration were posterior capsular-bag opacification, choroidal effusion, and dry age-related macular degeneration.

### 3.5. Complications and Additional Procedures

Subconjunctival 5-FU injections were administered to nine patients (19.6%) in the P-Ex-PRESS group and nine patients (23.1%) in the P-Trab group (*P* = 0.216). The average dose of 5-FU was 7.0 ± 3.5 mg in the P-Ex-PRESS group (a mean of 1.4 injections per patient) and 8.5 ± 2.3 mg in the P-Trab group (a mean of 1.7 injections) (*P* = 0.105). Needling was used in 11 patients (23.9%) in the P-Ex-PRESS group and in 12 in the P-Trab (30.8%) group (*P* = 0.305). One patient from the P-Ex-PRESS group (2.1%) and two patients from the P-Trab group (5.1%) (*P* = 0.462) underwent laser suturolysis. One patient from the P-Ex-PRESS group (2.3%) was fitted with an additional sealing suture (*P* = 0.354). Two patients from the P-Ex-PRESS group (4.6%) underwent reoperation: one due to extrusion of the mini seton through the scleral flap, and the other due to fibrosis of the filtering bleb. In both cases, classical trabeculectomy was performed. These patients were excluded from the study after the additional surgery, but their previous results were not excluded from the database. One patient from the P-Trab group (2.6%) underwent reoperation due to unsatisfactory IOP regulation. Two patients from the P-ExPress group (4.3%) and one (2.6%) from the P-Trab group had symptoms of malignant glaucoma that occurred at different time-points after surgery and were managed successfully with cycloplegics and compression dressing. The exact rate of complications is shown in Table 5.

### 3.6. Refractive Analysis of Astigmatism

**Figure 1:**
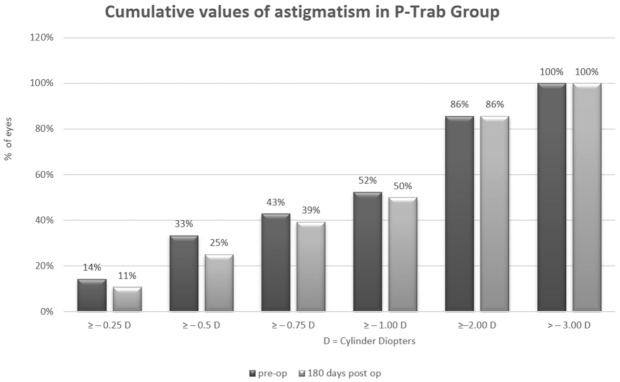
Cumulative values of astigmatism in the P-Trab group.

**Figure 2:**
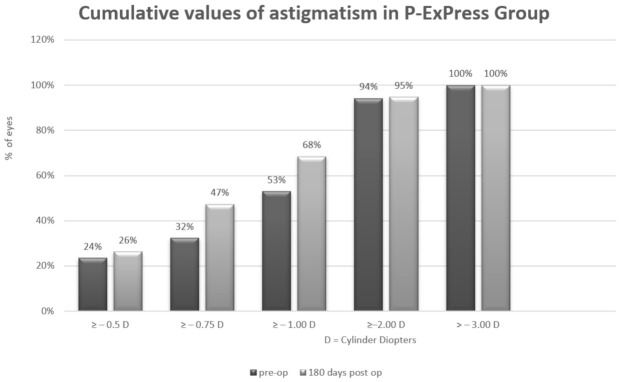
Cumulative values of astigmatism in the P-Ex-PRESS group.

Arithmetic analysis revealed an astigmatism magnitude of approximately 1 Dcyl in both groups for all analyzed periods. For P-Ex-PRESS, the magnitude was 1.15 ± 0.76 D preoperatively and 0.89 ± 0.52 D postoperatively. For P-Trab, this was 1.13 ± 0.93 D preoperatively and 1.20 ± 0.74 D postoperatively. No differences were found in the magnitude of astigmatism throughout the observation period or between groups (Table 6 and Table 7, Figure 1 and Figure 2).

### 3.7. Vector Analysis of Astigmatism

**Figure 3:**
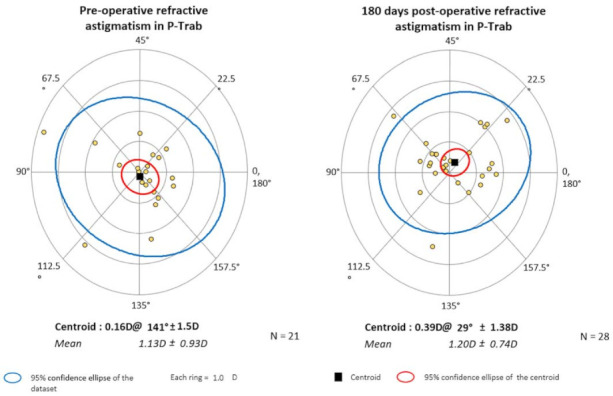
The mean values of astigmatism, with the axes, presented in centroid form on double angle plots for the P-Trab group (The black square indicates the centroid. The red circle indicates the 95% confidence ellipse of the centroid. The blue circle indicates the 95% confidence ellipse of the dataset. Yellow dots indicate individual values of astigmatism. Each ring indicates 1.0 D. Astigmatism trend in P-trab is oblique before and after the surgery).

**Figure 4:**
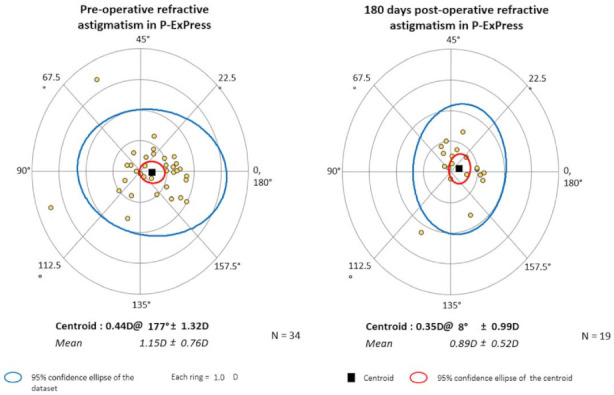
The mean values of astigmatism, with the axes, presented in centroid form on double angle plots for the P-Ex-PRESS group (The black square indicates the centroid. The red circle indicates the 95% confidence ellipse of the centroid. The blue circle indicates the 95% confidence ellipse of the dataset. Yellow dots indicate individual values of astigmatism. Each ring indicates 1.0 D. In P-Ex-PRESS astigmatism trend it is against the rule before and after surgery.

The mean values of astigmatism and the corresponding axes are presented numerically in Table 7 and graphically in centroid form on double-angle plots in Figure 3 and Figure 4.

## 4. Discussion

To the best of our knowledge, this is the first prospective study to compare P-Trab and combined phaco-Ex-PRESS device implantation based on SIA in glaucoma patients. Surprisingly, despite the use of different surgical techniques, the procedures did not differ when determining SIA. We also evaluated the postoperative complications of surgery and analyzed the IOP-lowering effect of combined surgeries.

In this study, astigmatism was of the ATR type in the P-Ex-PRESS group, and the direction had not changed by the 6-month follow-up. Astigmatism values changed slightly during the follow-up period, dropping from an average of 1.15 D to 0.89 D (*P* > 0.05). In the analyzed P-Trab group, mean astigmatism was 0.86 D before surgery and increased to 1.26 D after surgery (*P* > 0.05). Both before and after surgery, the direction of astigmatism was oblique.

Our study showed that despite the use of different surgical instruments and the discrepancy in the extend of tissue excision, astigmatism does not differ between the groups. These findings may confirm the assertion of Hugkulstone [6] that the astigmatism shift originates from a surgically induced gape around the scleral flap or the number of flap sutures (which were the same in both groups) and not from the removal of tissue under the scleral flap that leaves the corneal edge unsupported (which differed our groups); however, owing to the elasticity of the human sclera, this might be irrelevant for postoperative BCVA.

Our results contradict the findings of Tanito et al., who found in a retrospective study significant differences in SIA occur due to trabeculectomy and those due to ExPress implant trabeculotomy ab externo and microhook ab interno trabeculotomy. As expected, they found the least SIA after the minimally invasive procedures [14].

To date, the results of previous studies on the magnitude and direction of SIA are inconsistent. Hammel et al. [15] analyzed changes in corneal curvature with a Pentacam after implanting an Ex-PRESS [14]. On the first postoperative day, the anterior corneal astigmatism increased from 2.6 ± 3.3 to 4.7 ± 3.1 D (*P* = 0.19), but the curvature of the posterior cornea also changed (0.4 to 0.9). However, the changes were not statistically significant after 3 months. They observed a non-significant correlation between the increase in astigmatism and the presence of a low IOP due to anti-glaucoma surgery, which was also previously reported by Razeghinejad [16]. Hornová reported that ATR astigmatism was 0.8 D at 6 months after trabeculectomy [17]. Claridge et al. found statistically insignificant ATR astigmatism in some patients and statistically significant WTR in others [9]. They showed that the major change in the astigmatism vector was 1.25 ± 1.08 after 6 months in the vertical meridian. The authors assumed that vertical steepening was due to significant tissue contraction caused by excessive cauterization of the sclera. They also suspected that a large filtration cushion and postoperative ptosis could provoke vertical corneal steepening.

Law et al. compared the refractive results after P-Trab and phaco alone [18]. No differences between the expected and achieved refraction after surgery were observed, despite changes in corneal curvature and eyeball length. In their study, the average size of the cylinder after anti-glaucoma surgery was 1.31 ± 0.86 D, which was slightly larger than that in the control group, where the induced astigmatism was 0.44. There was a tendency towards short-sightedness, caused by shortening the eyeball length after trabeculectomy, as well as after implantation of drainage valves [19].

Cunliffe et al. performed tests on 19 eyes after trabeculectomy and evaluated the refraction and keratometry data [20]. Their findings included a reduction in the vertical corneal radius, and therefore a trend of astigmatism towards WTR in the early postoperative period. They proposed that the change in astigmatism was due to sclerostomy healing, causing the corneal edge to contract during trabeculectomy, resulting in a reduction of the horizontal corneal radius. After 2 months, the corneal radius returned to its preoperative value, probably due to a decrease in suture tension on the scleral flap. In addition, they noticed changes in anterior segment parameters, with the refraction shift toward myopia reaching 2 D. The hypothesis of a decrease in suture tone on the scleral flap does not coincide with the observations reported by Lima et al. [21], who did not observe an effect of laser suturolysis on the corneal curvature; however, they observed a shift in astigmatism in the direction of WTR of between 1.5 and 2.5 D, which persisted up to 3 months after surgery. They reported that changes in keratometry were underestimated compared to topography findings.

Our study has a few limitations. First, the data used for calculations were from autorefractometry and represented whole astigmatism, including lenticular astigmatism preoperatively and IOL postoperatively. In contrast, clinicians are concerned about overall astigmatism because it affects postoperative visual acuity. Second, a comparative study with keratometry could not be conducted, because the keratometry data were incomplete. Third, staff that performed the follow-up visits was not blinded; BCVA and IOP were measured with knowledge of the surgery that had been performed. Another limitation is the 6-month follow-up time. Existing data on astigmatism after anti-glaucoma surgery is inconsistent. According to one study, changes in corneal curvature after surgery can last up to 12 months [6]. Other studies have reported that such changes disappear after 6 months [7], and others found that refraction was constant after 2 to 3 months [22].

In future studies, blinding of all investigators could improve the reliability of the outcome data. Also, cornea mapping should be obtained before and after surgery to permit an exact assessment of the astigmatism. Another issue for future investigations is assessment of the possible impact of the Ex-PRESS device (among the other implants) on endothelial cell loss.

## 5. Conclusions

In conclusion, the P-Ex-PRESS and P-Trab procedures did not differ in inducing postoperative astigmatism or refractive errors; the astigmatism trend noted during observation remained the same in both groups (ATR in P-Ex-PRESS and oblique in P-Trab). Comparable results were found in the groups for final visual acuity. The two procedures showed similar antihypertensive efficacy in treating open-angle glaucoma. This study confirms the noninferiority of the Ex-PRESS device, which is relevant when making optimal clinical decisions with patients, as Ex-PRESS is comparatively less invasive than trabeculectomy.

## Figures and Tables

**Table 1:** Patient demographic data.

Group.	P-ExPress	P-Trab	*P* *
Follow-up (months)	6.7 ± 0.4	6.9 ± 0.5	0.249
Number	43	38	-
Age (years)	72.1 ± 4.62	67.2 ± 9.28	0.374
Sex (female/male)	26/17	28/10	0.579
Eye (right/left)	23/20	17/21	0.658
Glaucoma Type			
POAG	27	21	0.482
PEX	14	17	
Pigmentary	2	0	
LOCS III scale (NC_1_/NC_2_/NC_3_)	12/23/6	11/20/7	0.768

LOCS III: lens opacities classification system III; P-ExPress: phaco-ExPress group; P-Trab: phacotrabeculectomy group; * Student’s *t*-test or x^2^ test; POAG: primary open-angle glaucoma; PEX: pseudoexfoliation glaucoma.

**Table 2:** Intraocular pressure (IOP) mean values, median values, standard deviations, and range in the phaco-Ex-Press (P-ExPress) and phaco-trabeculectomy (P-Trab) groups at specific times after surgery.

Time	P-ExPress			P-Trab			*P* *
	Mean (SD)	Median	Range	Mean (SD)	Median	Range	
Pre-op	25.3 ± 7.1	25.00	12–50	26.8 ± 11.3	25.00	12–62	0.877
1st day	16.7 ± 8.0	16.00	4–29	15.7 ± 7.1	16.00	3–31	0.867
7th day	15.2 ± 6.6	14.00	7–23	16.9 ± 4.8	18.00	3–19	0.236
1st month	17.6 ± 10.2	16.00	8–27	17.3 ± 8.2	16.00	10–26	0.653
3rd month	14.9 ± 3.2	16.00	5–23	14.5 ± 4.1	15.00	7–24	0.645
6th month	15.1 ± 4.3	15.00	7–22	15.9 ± 2.9	16.00	8–23	0.281

P-ExPress: phaco-Ex-Press group; P-Trab: phacotrabeculectomy group; Pre-op: pre-operatively; SD: standard deviation; * Mann-Whitney U test.

**Table 3:** Number of hypotensive drugs: mean values, median values, standard deviations, and ranges in the phaco-Ex-Press (P-ExPress) and phaco-trabeculectomy (P-Trab) groups before and 6 months after surgery.

Time	P-ExPress			P-Trab			*P* *
	Mean (SD)	Median	Range	Mean (SD)	Median	Range	
Pre-op	2.91 ± 0.9	3.0	2–4	3.26 ± 0.8	3.0	3–4	0.79
6 mo.	0.46 ± 1	0	-	1.39 ± 1.2	2	0–2	0.3

Pre-op: pre-operatively; mo: months; * Mann–Whitney U test.

**Table 4:** Visual acuity (logMAR) mean values, median values, standard deviations, and ranges in the phaco-ExPress (P-ExPress) and phaco-trabeculectomy (P-Trab) groups before and 6 months after surgery.

Time	P-ExPress			P-Trab			*P* *
	Mean (SD)	Median	Range	Mean (SD)	Median	Range	
Pre-op	0.53 ± 0.55	0.30	0–2.4	0.48 ± 0.5	0.16	0–2	0.68
6 mo.	0.22 ± 0.42	0.1	0–1.7	0.17 ± 0.28	0.05	0–1.4	0.57

mo: months; Pre-op: pre-operatively; SD: standard deviation; * Mann–Whitney U test.

**Table 5:** Complications that were observed in the participants.

	P-ExPress *n* (%)	P-Trab *n* (%)	*P* *
Intraoperative			
Bleeding	-	1 (2.6)	0.645
Postoperative			
Hyphema			
blood level in AC	1 (2.1)	1 (2.6)	0.875
erythrocytes in AC	-	-	-
Wound leakage	3 (6.5)	1 (2.6)	0.391
Fibrosis	9 (19.6)	9 (23.1)	0.784
Anterior chamber cells	3 (6.5)	3 (7.7)	0.834
Hypotony			
until 7 days	-	2 (5.1)	0.115
until 30 days	-	1 (2.6)	0.411
until 180 days	1 (2.1)	1 (2.6)	0.896
Choroid detachment	1 (2.1)	3 (7.7)	0.231
Macular edema	1 (2.1)	-	0.354

AC: anterior chamber; P-ExPress: phaco-Express group; P-Trab: phaco-trabeculectomy, group; * x^2^ test.

**Table 6:** Autorefractometry data: mean magnitude of positive cylinder form in the phaco-ExPress (P-ExPress) and phaco-trabeculectomy (P-Trab) groups before and 6 months after surgery.

Time	P-ExPress			P-Trab			*P* *
	Mean	Median	SD	Mean	Median	SD	
Pre-op	1.15	1.0	0.76	1.13	0.75	0.93	0.175
6 mo.	0.89	0.87	0.52	1.20	1.06	0.75	0.2

mo: months; Pre-op: pre-operatively; * Mann–Whitney U test.

**Table 7:** Centroids (mean astigmatism with its axis) and direction of astigmatism in the phaco-ExPress (P-ExPress) and phaco-trabeculectomy (P-Trab) groups before and 6 months after surgery.

Time	P-ExPress			P-Trab			*P* *
	Centroid	Axis	Trend	Centroid	Axis	Trend	
Pre-op	0.44	177.42	ATR	0.16	140.61	OBLIQUE	0.5
6 mo.	0.35	7.71	ATR	0.39	29.47	OBLIQUE	0.38

ATR: Against-the-rule; * Mann–Whitney U test.

## Data Availability

All materials and information are available upon e-mail request to the corresponding author. The names and exact data of the study participants may not be available because of privacy policies.
